# Noble Metal-Assisted Surface Plasmon Resonance Immunosensors

**DOI:** 10.3390/s20041003

**Published:** 2020-02-13

**Authors:** Jin-Ha Choi, Jin-Ho Lee, Joohyung Son, Jeong-Woo Choi

**Affiliations:** 1 Department of Chemical and Biomolecular Engineering, Sogang University, Seoul 04107, Korea; jinhachoi@sogang.ac.kr (J.-H.C.); organoid@sogang.ac.kr (J.S.); 2 School of Biomedical Convergence Engineering, Pusan National University, Yangsan 50612, Korea; leejh@pnu.ac.kr; 3 Department of Biomedical Engineering, Sogang University, Seoul 04107, Korea

**Keywords:** surface plasmon resonance, noble nanoparticle, immunosensor, label-free detection, biosensor

## Abstract

For the early diagnosis of several diseases, various biomarkers have been discovered and utilized through the measurement of concentrations in body fluids such as blood, urine, and saliva. The most representative analytical method for biomarker detection is an immunosensor, which exploits the specific antigen-antibody immunoreaction. Among diverse analytical methods, surface plasmon resonance (SPR)-based immunosensors are emerging as a potential detection platform due to high sensitivity, selectivity, and intuitive features. Particularly, SPR-based immunosensors could detect biomarkers without labeling of a specific detection probe, as typical immunosensors such as enzyme-linked immunosorbent assay (ELISA) use enzymes like horseradish peroxidase (HRP). In this review, SPR-based immunosensors utilizing noble metals such as Au and Ag as SPR-inducing factors for the measurement of different types of protein biomarkers, including viruses, microbes, and extracellular vesicles (EV), are briefly introduced.

## 1. Introduction

The optoelectronic phenomenon of surface plasmon resonance (SPR), which is widely utilized in optical biosensors, was established from studies involving excitation of surface plasmons on metallic surfaces, especially noble metals [1,2,3,4]. When metallic surfaces are exposed to light, a photon is trapped near the metallic surface and prompts the electrons to move as a single electrical entity, which is known as surface plasmon (SP). This oscillation of electrons in a metal film (PSPR, propagating surface plasmon resonance) leads to the formation of an electromagnetic field that exponentially decays out from the surface, also known as the evanescent field [5,6]. Differently, when a surface plasmon is confined on nanomaterials, this unique physical property is highly localized around the nanoparticle, leading to a non-propagating localized surface plasmon with a specific frequency (LSPR, localized surface plasmon resonance) [7,8]. Although these optical phenomena have attracted interest in various fields, use is most prominent in the field of biosensor development [9,10,11,12,13]. 

Both PSPR and LSPR-based biosensors utilize refractive index changes to sensitively detect mass changes based on the molecular interaction that occurs on the surface of a metal film or nanoparticle [14,15,16,17]. For example, in the case of a planar surface, the binding of bio/chemical molecules perturbs the plasmon and leads to a shift of the resonance angle of the incoming photons [18]. Thus, no additional labeling materials are required to transduce the binding event, and the method also provides quantitative real-time measurements with remarkable sensitivity over a broad range of bio/chemical molecules [19]. Although PSPR-based sensors is known to have a much higher refractive index sensitivity compare to LSPR-based sensors, as the sensitivity of PSPR-based sensor is highly affected by the penetration depth of evanescence field (ca. 200 nm) [20,21,22], their sensitivity towards bio/chemical molecular interaction is similar [13]. However, due to the much shorter electromagnetic decay length on the nanoparticles compared to gold films, which allow confining the response to a smaller sensing volume, LSPR-based sensor is known to be more sensitive to small molecular binding and less sensitive to bulk effects [23]. Considering these advantages, both PSPR- and LSPR-based analytical methods have been extensively utilized to investigate bio/chemical molecular interactions for diagnostic purposes. 

PSPR- and LSPR-based biosensors commonly use antibodies as a bio-receptors for recognition of the target of interest. Recent approaches have been extensively applied to utilize new bio-receptors such as aptamers and imprinted polymers for replacement of antibodies; however, antibody-based assays are still considered the primary choice for developing PSPR- and LSPR-based biosensors, owing to their superior affinity, selectivity, and stability [24,25]. To this end, a wide range of antibody immobilization methods has been developed to achieve optimal consistency while maintaining the activity of immobilized antibodies. The formation of well-ordered interfaces is also considered as an essential aspect to achieve a reliable and sensitive sensor platform [24].

In this review, we will provide a selective overview of the recent advances in the development of highly sensitive PSPR- and LSPR-based immunosensors as excellent clinical diagnostic systems. Sections will focus on protein biomarkers, viral agents, microbes, and extracellular vesicles as selective biomarkers. Finally, the future perspective of the development of SPR- and LSPR-based analytical methods such as miniaturization will be discussed. We hope that this review will provide brief and concise information on the development of SPR- and LSPR-based biosensors and emphasize research from various disciplines for further development and improvement of SPR- and LSPR-based analytical methods for more significant biomedical applications.

## 2. Analytical Methods for Protein Biomarkers 

**Figure 1:**
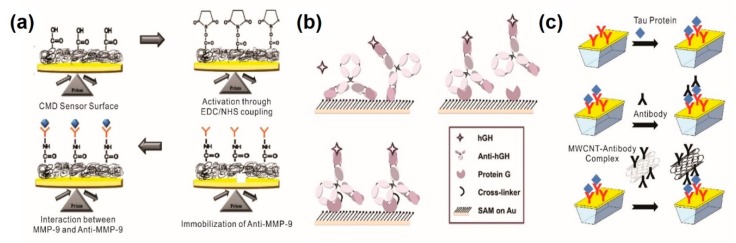
Propagating surface plasmon resonance (PSPR)-based immunosensors for protein biomarker detection. (**a**) Detection of human matrix metalloproteinases-9 by SPR-based immunosensor; (**b**) signal enhancement of SPR-based immunosensor by protein G meditated site direct immobilization of antibody; (**c**) signal amplification of SPR-based immunosensor by utilizing multi-walled carbon nanotubes as a mass enhancer. (**a**) Figure reproduced with permission from [28], © 2016 Elsevier; (**b**) Figure reproduced with permission from [29], © 2015 RSC Publishing; (**c**) Figure reproduced with permission from [30], © 2017 Elsevier.

Proteins in biological fluids are promising candidates as indicators of disease risk and allow for early diagnosis for more effective treatment. Taking advantage of PSPR and LSPR-based analytical methods has created extensive applications in the development of immunoassays for diagnosing diseases with protein biomarkers [26,27]. Mohseni et al. have reported on the development of SPR-based immunosensors for real-time and label-free detection of recombinant human matrix metalloproteinases-9 (MMP-9) as a biomarker for malignant tumor progression and metastasis [28] (Figure 1a). A carboxymethyl dextran hydrogel sensor chip was utilized for the immobilization of anti-MMP-9 monoclonal antibodies through amine coupling reagents and further experiments. Through optimization of parameters (e.g., equilibrium dissociation constant (KD) and maximum binding capacity (Rmax)) that affect the SPR-based sensing property, the limit of detection (LOD) was found to be 8 pg/mL for MMP-9 in saliva samples with linearity in the range of 10~200 ng/mL. 

As the SPR signal is based on the molecular interaction of a receptor (antibody) and a target analyte (antigen), Makaraviciute et al. have shown that meditating protein G for site-directed antibody immobilization can amplify the analytical signal 3.5 times higher than randomly oriented antibodies [29] (Figure 1b). Typically, random immobilization can achieve higher surface coverages while site-directed immobilization provides better sensitivities [31]. Based on these findings, authors have detected human growth hormone (hGH) at a LOD of 21.9 ng/mL, with a linear detection range from 66.4~199 ng/mL. However, due to the small mass of a protein molecule, one of the foremost methods to enhance the SPR-based sensor response is the employment of additional materials as high mass labels [32]. For this purpose, Lisi et al. modified multi-walled carbon nanotubes (MWCNTs) and decorated them with the secondary antibody as a mass enhancer to amplify the signal for Tau protein detection [30] (Figure 1c). Functionalization of antibodies with MWCNTs significantly enhances sensor response up to 10^2^ fold, which was challenging to obtain with the protein analyte itself or by employing conventional unconjugated sandwich assays [30]. Similarly, Pawula et al. utilized gold nanoparticles (AuNPs) as a mass enhancer to amplify the sandwich assay-based SPR immuno-sensor response for cardiac troponin T (cTnT) detection [33].

**Figure 2:**
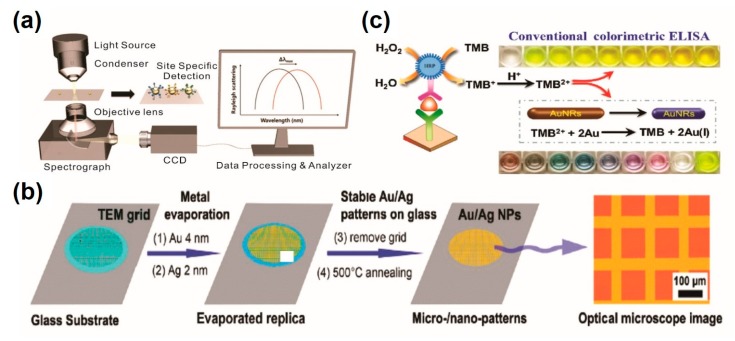
Localized surface plasmon resonance (LSPR)-based immunosensors for protein biomarker detection. (**a**) Selective recognition of multiplex cancer biomarkers by LSPR immunosensor; (**b**) signal enhancement of LSPR-based immunosensor by employing bimetallic nanostructures; (**c**) LSPR band shift based on the GNR etching resulted by TMB reaction. (**a**) Figure reproduced with permission from [37], © 2015 Elsevier; (**b**) Figure reproduced with permission from [38], © 2014 American Chemical Society; (**c**) Figure reproduced with permission from [39], © 2017 Elsevier.

In a different approach, the LSPR mechanism has been utilized for the development of analytical methods for the detection of protein biomarkers [34,35,36]. In typical LSPR-based analytical methods, the bio/chemical interaction on the surface of metallic nanoparticles leads to an increase of the refractive index of a local medium, resulting in a resonant wavelength shift. Based on this unique plasmon response of single AuNP and antibody-antigen binding activity, Lee et al. described label-free multiplex detection of cancer biomarkers including α-fetoprotein (AFP), carcinoembryonic antigen (CEA), and prostate-specific antigen (PSA) [37] (Figure 2a). The LSPR-based plasmonic biosensor was fabricated by immobilizing AuNPs on a hydrophilic-hydrophobic patterned glass slide in a site-specific manner, and it functionalized antibodies that selectively recognize target proteins. The binding activity between the antibody and antigen (target) was monitored through spectral changes resulting from the local refractive index of individual AuNPs. Owing to the outstanding properties of the LSPR mechanism, the proposed platform exhibits excellent selectivity and sensitivity with a LOD of 6.28 pg/mL, 16.9 pg/mL and 284 fg/mL for AFP, CEA, and PSA, respectively, from patient-mimicked serum. As the intrinsic refractive index sensitivity is highly affected by the frequency-dependent dielectric function, size, and shape of materials, Jia et al. have demonstrated that Au/Ag bimetallic NPs could preserve a sharper plasmonic peak with more sensitive plasmonic responses, compared to monometallic AuNPs [38] (Figure 2b). Two sequential evaporations of gold and silver on a glass substrate, followed by annealing, resulted in a uniform size and shape distribution of bimetallic NPs. As proof of concept, bovine serum albumin (BSA) was applied as a model protein marker and a LOD of 0.01 ng/mL was observed under the optimized conditions.

Conversely, Ma et al. demonstrated that the LSPR peak shift could also occur through structural changes of noble-metal nanostructures [39]. Authors have described that the product (TMB2+) of horseradish peroxidase (HRP)-catalyzed oxidation of 3, 3′, 5, 5′-tetramethylbenzidine (TMB) can etch out the gold nanorods (GNRs), which generates vivid color responses based on the LSPR peak shift through structural changes [39] (Figure 2c). Based on this finding, researchers have utilized GNRs in a commercially available HRP-TMB immunoassay system for visual quantification of CEA and PSA with the naked eye. Similarly, the SPR and LSPR phenomena have been widely utilized in the development of different types of analytical methods for the determination of protein biomarkers [40]. Recent research on SPR- and LSPR-based analytical methods for protein marker detection are compared in Table 1. 

## 3. Analytical Methods for Viral Agents

A sensitive and selective analytical method for pathogenic viral agents is also critical for the successful diagnosis and treatment of diseases [41]. Most of the diseases caused by viruses such as flu and the common cold can be self-recovered by the innate immune response; however, some viruses evade this mechanism and cause life-threatening diseases such as Ebola, dengue hemorrhagic fever, and acquired immune deficiency syndrome [42,43,44]. Conventional methods for diagnosing infection with viral agents, such as serologic tests and enzyme-linked immunosorbent assays (ELISA), are not sensitive enough and are time-consuming [45,46]. To this end, SPR has gained interest from the medical field for the development of highly sensitive and selective analytical methods for viral agents. 

**Figure 3:**
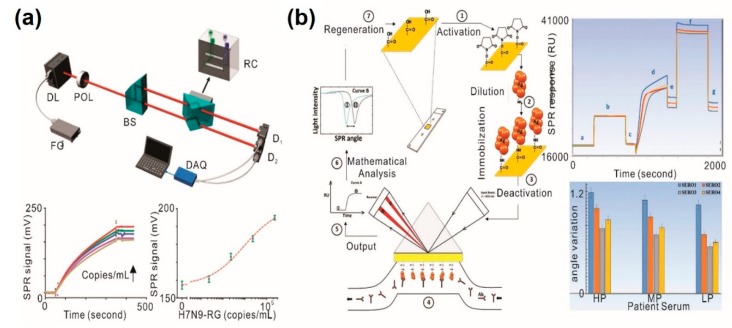
SPR-based immunosensors for viral agents detection. (**a**) Detection of Avian Influenza A H7N9 Virus by SPR-based immunosensor; (**b**) selective recognition of four different serotypes of dengue virus by SPR-based immunosensor from clinical samples. (**a**) Figure reproduced with permission from [47], © 2018 American Chemical Society; (**b**) Figure reproduced with permission from [48], © 2014 Springer Nature.

Chang et al. developed an intensity-modulated surface plasmon resonance (IM-SPR)-based immunosensor as a label-free, rapid test for avian influenza A H7N9 [47] (Figure 3a). To maximize sensitivity, authors employed a newly developed antibody which specifically recognizes the H7N9 virus but no other clinical human influenza isolates. By incorporating a highly specific antibody, a 20-fold increase in sensitivity, as compared to ELISA, was observed in less than 10 min. The detection limit in samples spiked with nasal mucosa from flu-like syndrome patients was observed to be 402 copies/mL, which is significantly lower than the detection limit of conventional influenza detection approaches including rapid influenza diagnostic tests and quantitative reverse transcription-polymerase chain reaction (qRT-PCR). Similarly, Loureiro et al. developed a PSPR-based immunoassay for the rapid diagnosis of dengue viral infection [49]. In this method, a neutravidin-biotin mediated monoclonal antibody is immobilized on a thin gold film as the sensing element. The binding interaction between the monoclonal antibody and the dengue virus (DENV) results in a pronounced thickness change that is optically recorded in real-time using an integrated microfluidic setup. The developed method is applicable even when testing complex biological fluids composed of non-specific binding interferences. The experimental LOD was estimated to be 2 × 10^4^ particles/mL. Jahanshahi et al. also developed a PSPR-based dengue diagnostic test by targeting the DENV immunoglobulin M (IgM) [48] (Figure 3b). The authors immobilized the four different dengue virus serotype antigens on a biochip surface as ligands instead of immobilizing DENV-specific antibodies. Note that, here the antibody was considered as a target instead of antigen. The SPR angle change clearly showed variation between patient serum antibody titers categorized as high positive (HP), mid positive (MP) and low positive (LP). Each dengue virus serotype has a linear slope variation on the SPR angle, which allows for the distinction of HP, MP, and LP titers. Furthermore, samples of tick-borne encephalitis (negative dengue NS1) and hepatitis C (negative dengue IgM) viruses were tested to ensure the high sensitivity and specificity of the proposed method. Consequently, the ratio of each dengue serotype in the samples was able to be determined with 83%~93% sensitivity and 100% specificity.

**Figure 4:**
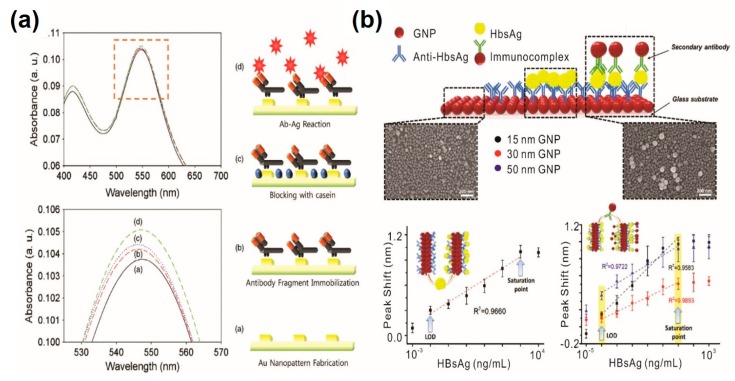
LSPR-based immunosensors for viral agent detection. (**a**) Detection of HIV-1 virus by LPSR-based immunosensor; (**b**) signal amplification of LSPR-based immunosensor by forming hetero-assembled gold nanoparticles based on the sandwich assay. (**a**) Figure reproduced with permission from [51], © 2013 Elsevier, (**b**) Figure reproduced with permission from [52], © 2018 Elsevier.

A few studies have also reported on the development of analytical methods for viruses based on the LSPR mechanism as well. Luo et al. developed a novel immunosensor to detect Newcastle disease virus (NDV) by integrating excessively tilted fiber grating (Ex-TFG) coated with AuNP [50]. Owing to the local surface plasmon resonance (LSPR) effect of the AuNP, the detection limit and sensitivity of the proposed analytical method was approximately 5~10 times improved compare to conditions without AuNP treatment. By monitoring the resonance wavelength shift, the LOD was estimated as ~25 pg/mL. Similarly, Lee et al. have proposed a simple analytical method for HIV virus-like particle detection based on the LSPR mechanism [51] (Figure 4a). Researchers fabricated highly ordered circular-shaped Au nanopatterns on a transparent indium tin oxide substrate through an electrochemical deposition method and utilized it for HIV detection. The presence of HIV was characterized through absorbance shifts resulting from changes in the refractive index on the surface of Au nanopatterns without any additional labeling materials. Furthermore, Kim et al. integrated hetero-assembled AuNPs by the sandwich assay method to detect the hepatitis B surface antigen (HBsAg) in a more sensitive manner [52] (Figure 4b). By forming a hetero-assembled sandwich-immunoassay, the LOD was improved up to 100 fg/mL of HBsAg, while a single-layered LSPR-based analytical device using AuNPs was able to detect as low as 10 pg/mL. As can be seen, both PSPR and LSPR-based analytical methods are widely utilized for the sensitive and selective detection of pathogenic viral agents. Recent research on SPR- and LSPR-based analytical methods for viral agent detection are compared in Table 2.

## 4. Analytical Methods for Pathogenic Microbes

Several pathogenic microbes critically threaten humans as a result of ease of infection and long latency periods [53]. Therefore, pathogenic microbes such as bacteria are important targets for early diagnosis and treatment in medicine, public health, and food safety [54]. Infectious diseases are the leading cause of disease and death worldwide, with millions of casualties each year. In particular, infectious diseases from bacteria are most challenging in low-income countries [55,56]. Representatively, *Escherichia coli* (*E. coli*) O157:H7 and *Salmonella* are the leading causes of bacterial diseases [53,57]. The primary cause of mortality is an inaccurate and time-consuming diagnosis of bacterial infection. Therefore, the development of more specific and sensitive analytical platforms that can be employed at the point of care are urgently needed. For bacterial detection, immunoreaction-based assays including enzyme-linked immunosorbent assays (ELISAs) are usually utilized to detect particular surface proteins [58,59,60]. A variety of surface antigens present on a cell surface can bind to a specific antibody, and the expressed antigen can vary depending on the type of bacteria. Therefore, immune-based analytical methods could be applied to detect bacteria and diagnose a bacterial infection. However, the conventional immunoassay is time-consuming and costly due to the specialist technical staff and equipment required. Among recent strategies for more efficient detection of bacteria, SPR-based bacterial sensors have been developed [61,62]. Previously, we developed SPR-based biosensors for bacterial detection using the specific immune reaction between a surface antigen on the bacteria and a specific antibody on the Au plate [63,64]. For the oriented immobilization of antibodies, protein G was pre-immobilized on the Au surface. The pathogen binding of the antibody-immobilized Au plate was determined by SPR spectroscopy. In result, four different pathogenic microbes, including *Escherichia coli (E.Coli) O157:H7, Salmonella Typhimurium, Legionella pneumophila, and Yersinia enterocolitica* could be selectively detected with high efficiency. In addition, *Vibrio cholerae (V. cholerae)* O1 was successfully measured by SPR analysis on an 11-mercaptoundecanoic acid (MUA) and antibody-immobilized Au plate. The detection range was between 10^5^ and 10^9^ cells/mL. Since then, various types of SPR-based biosensing platforms have been developed for bacterial detection. In this section, recently developed pathogenic bacteria detection systems will be introduced briefly.

**Figure 5:**
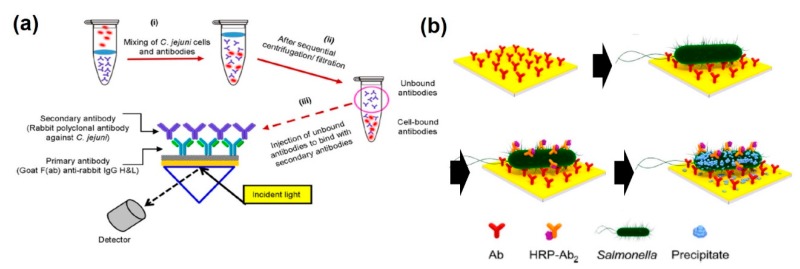
PSPR-based immunosensors for pathogenic microbe detection. (**a**) The specificity of developed C. jejuni assay using unbounded secondary antibody detection; (**b**) SPR chip with the immobilized capture antibody, binding of Salmonella, HRP-Ab conjugate, and HRP-catalyzed conversion of 4-chloro-1-naphthol to insoluble benzo-4-chlorocyclohexadienone. (**a**) Figure reproduced with permission from [68], © 2019 Springer Nature; (**b**) Figure reproduced with permission from [69], © 2016 American Chemical Society.

As described above, several pathogenic microbes have been measured by SPR-based immunoassay, which utilizes a noble metal such as Au as a plasmonic effector. Taheri et al. developed a sensitive *V. cholerae* detection system using antibodies against recombinant outer membrane protein (anti-OmpW) [65]. The high-affinity interaction between anti-OmpW and OmpW (K_D_ = 2.4 × 10^−9^ M) induced sensitive detection of *V. cholerae*, with a detection limit of 43 cells/mL and a high R^2^ value (>0.98). Makhneva et al. demonstrated the use of a plasma polymer-functionalized Au surface for effective antibody immobilization [66]. An anti-*Salmonella* antibody was successfully functionalized on an Au surface and the bacteria was detected at a level as low as 10^5^ CFU/mL with a wide linear response by both SPR and quartz crystal microbalance (QCM) analytical methods simultaneously. Chen et al. developed a singleplex immunoassay for *Salmonella* serotypes (*S. Enteritidis, S. Heidelberg*, and *S. Typhimurium*) to evaluate the potential of SPR imaging in specific pathogenic bacteria detection [67]. LODs were found to be 2.1 × 10^6^ CFU/mL in buffer solution and 8.9 × 10^7^ CFU/mL of a microflora mixture. Masdor et al. found that *Campylobacter jejuni* was sensitively detected by an SPR analytical method using a subtractive inhibition assay (SIA), which measured the unbound anti-*Campylobacter jejuni* from the supernatant [68] (Figure 5a). This SIA-based SPR immunosensor exhibited outstanding sensitivity with a LOD of 131 ± 4 CFU/mL and a 95% confidence interval of 122 to 140 CFU/mL, with high specificity. It could detect the minimum infectious dose of *Campylobacter jejuni* (below 500 CFU/mL), making the method suitable as a rapid and sensitive method for the early detection of microbe infection. For signal enhancement and sensitive detection of pathogenic microbes, Farka et al. described a label-free detection system for *Salmonella Typhimurium* at levels as low as 10^4^ CFU/mL within 10 min [69] (Figure 5b). In addition, a horseradish peroxidase (HRP)-labeled secondary detection antibody was specifically attached to *Salmonella Typhimurium*, and it produced precipitation of 4-chloro-1-naphthol to benzo-4-chlorocyclohexadienone. It could induce signal enhancement and result in a LOD of 100 CFU/mL with a linear range up to 10^6^ CFU/mL. 

**Figure 6:**
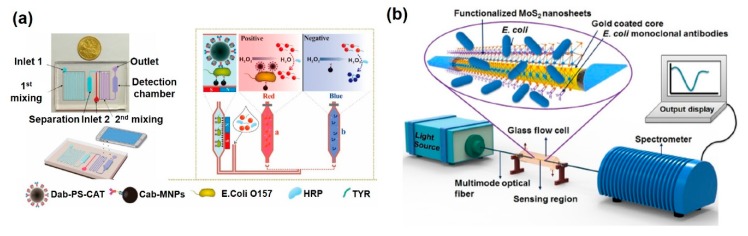
LSPR and optic fiber mediated SPR-based immunosensors for pathogenic microbe detection. (**a**) Colorimetric immunosensors with microfluidic device for the detection of E. coli O157 by aggregation of Au nanoparticles; (**b**) fiber optic SPR immunosensor based on MoS_2_ nanosheet for the detection of E. coli. (**a**) Figure reproduced with permission from [71], © 2019 Elsevier, (**b**) Figure reproduced with permission from [74], © 2019 Elsevier.

Noble metal nanostructures have been frequently used for microbe detection systems to enhance functions such as specificity and sensitivity. Haddada et al. developed an Au nanoparticle-antibody bioconjugate, which was engineered by covalently linking anti-staphylococcal enterotoxin A (SEA) antibodies, for a colorimetric assay by localized surface plasmon resonance (LSPR) [70]. SEA was measured in both the ng/mL and μg/mL working ranges with a 5 ng/mL LOD. In addition, it could be stored for 1 year at 4 °C without loss of detection performance. Zou et al. utilized Fe_3_O_4_@Au nanoparticles as a magneto-plasmonic nanoprobe for the amplification of SPR signals. The Fe_3_O_4_@Au nanoparticles can concentrate the electric charge density and widen the surface area of the antibody functionalization. The electronic coupling effect could be enhanced and the SPR signals amplified as high as 30-fold above the LOD. Using this system, tuberculosis caused by Mycobacterium tuberculosis was successfully detected at a LOD of 0.1 ng/mL. Zheng et al. developed a microfluidic biosensor with Au and magnetic nanoparticles for simple, rapid, and sensitive detection of *E. coli O157:H7* [71]. Two different anti-*E. coli* antibodies were functionalized on the magnetic nanoparticle and polystyrene microsphere, forming a sandwich structure with *E. coli O157:H7*. Aggregation of the Au nanoparticles was induced through the crosslinking of phenolic hydroxyl moieties in tyramine by catalase, which is catalyzing hydrogen peroxide. The resulting color change was measured using a smartphone imaging application to detect *E. coli O157:H7* with good specificity and sensitivity (50 CFU/mL of LOD) (Figure 6a). Zhang et al. showed a sandwich-immunoassay system for staphylococcal enterotoxin serotype A (SEA) by the color change of Au nanoparticles [72]. On a transparent slide glass, Au nanoparticles could be attached when SEA was captured, and the color appeared as red at the detection spot. The detection range was 10 to 50 ng/mL with a LOD of 1.5 ng/mL. Optical fiber with a coating of a noble metal such as Au or Ag is also frequently used for microbe detection. Arcas et al. reported on an SPR-based optical fiber biosensor for the detection of *E. coli* by immunoassay [73]. In this study, U-shaped plastic optical fiber was coated with Au for the induction of the SPR phenomenon. In 70 nm and 100 nm Au-coated optical fiber, the SPR effect is predominant, and it can be used to detect bacteria at concentrations as low as 1.5 × 10^3^ CFU/mL. Kaushik et al. developed a molybdenum disulfide (MoS_2_) nanosheets functionalized optic fiber to enhance the signal of an SPR immunosensor for the sensitive detection of E. coli [74] (Figure 6b). The two-dimensional nanosheet (MoS_2_) was anchored to the surface of the Au coated optic fiber and monoclonal antibodies were immobilized on the functionalized nanosheets via hydrophobic interactions. This label-free immunosensor displays better performance (detection limit 94 CFU/mL) and a higher sensitivity (2.9 nm/1000 CFU/mL; 3135 nm/refractive index unit (RIU)) than conventional optic fiber biosensors (detection limit 391 CFU/mL, sensitivity 0.6 nm/1000 CFU/mL, 1646 nm/RIU), with a rapid detection time (about 15 min). In this way, a noble metal-assisted nanostructure or optic fiber could improve the performances of SPR-based immunosensor such as sensitivity, selectivity and reduced assay time. The recent researches on SPR and LSPR-based analytical methods for bacterial detection are compared in Table 3. 

## 5. Analytical Methods for Extracellular Vesicles (EVs)

In animal cells, extracellular vesicles (EVs) are released by budding from the membrane of a mother cell and are transferred to other cells [75,76,77,78]. EVs can be produced inside the multivesicular endosome (MVE). When MVE is fused to the cell membrane, the secreted EVs are called exosomes, with sizes of <200 nm diameter and contain several biomolecules, such as proteins, mRNA, miRNA, and lipids, with signature characteristics of their mother cells [79,80]. Therefore, secreted exosomes can represent their mother cells, including the cell type, cell cycle, and stage of cancer. Particularly, exosomes play key roles in tumorigenesis and cancer progression, including immunosuppression, angiogenesis, and metastasis [78,81,82,83]. Based on these characteristics, exosomes are one of the best candidates to be used as potential biomarkers for noninvasive cancer. Cancer-associated antigens are highly enriched on the surface of exosomes from cancer cells. Therefore, utilizing their surface biomarkers is the most promising approach for the simple and rapid detection of cancers. For the development of biosensors, tetraspanins, such as CD9, CD63, CD81, and CD82, on the surface of exosomes are normally employed as targets for total exosome detection. On the other hand, specific proteins on the surface of exosomes such as carcinoembryonic antigen (CEA), epithelial cell adhesion molecule (EpCAM), human epidermal growth factor receptor 2 (HER2), insulin-like growth factor receptor (IGFR), latent membrane protein 1 (LMP1), melanoma cell adhesion molecule (MCAM), and prostate-specific membrane antigen (PSMA) can be utilized for the detection of cancer cells. In this section, SPR-based immunosensors will be discussed for the determination of exosome levels, which are related to several cancers. 

**Figure 7:**
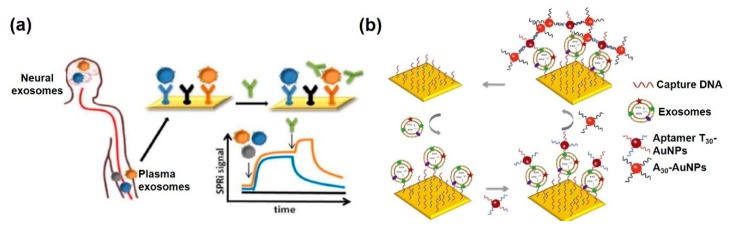
PSPR-based immunosensors for extracellular vesicle detection. (**a**) Detection and characterization of different brain-derived exosomes by SPR imaging; (**b**) dual Au nanoparticle-assisted signal amplification of SPR signals for determination of breast cancer-related exosomes. (**a**) Figure reproduced with permission from [84], © 2018 American Chemical Society; (**b**) Figure reproduced with permission from [86], © 2019 Elsevier.

SPR is a label-free, real-time analysis technique and is extremely sensitive to biological binding events occurring within 200 nm of wave depth of the Au layer. This distance directly matches the dimension of exosomes. Therefore, SPR-based biosensors are perfectly suited for the study of exosomes. The substrate used for reported SPR biosensors for exosome detection has been primarily based on Au-based plates. Picciolini et al. demonstrated the SPR imaging assay using an antibody array, which could bind to the surface protein of the exosome, on an Au plate for the separation and characterization of multiple exosomes from diverse neuronal cells [84] (Figure 7a). Exosomes from oligodendrocytes and neurons were measured with high sensitivity and selectivity. Subsequently, quantification of CD81 and GM1 (monosialotetrahexosylganglioside), exosome-specific proteins of each subpopulation, were successfully conducted by applying a second antibody on the exosome. These results verify the extreme inconsistency of exosome composition, even though the mother cells were of similar origin. Sina et al. reported a real-time, label-free detection method for breast cancer-related exosomes from complex biological samples using a SPR immunosensor [85]. Using this simple platform, HER2—specific exosomes were captured and detected on an anti-HER2-functionalized Au plate, at concentrations as low as 0.83 × 10^7^/mL. 

**Figure 8:**
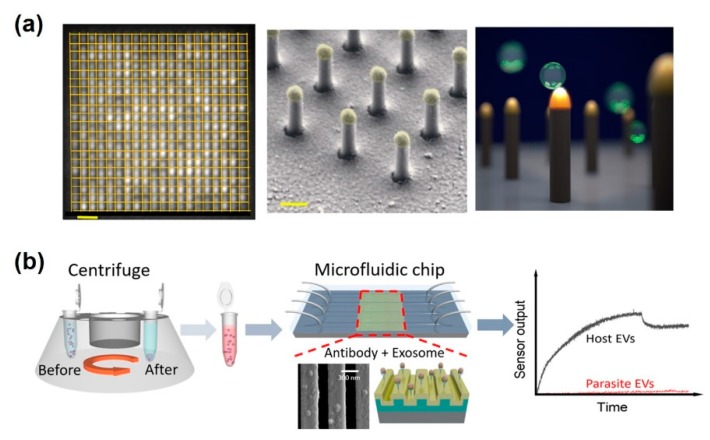
LSPR-based immunosensors for extracellular vesicle detection. (**a**) LSPRi sensor chip and high-magnification false-colored SEM image showing individual nanopillars, allowing digitized exosome detection. Scale bars are 1 μm and 200 nm, respectively; (**b**) microfluidic photonic crystal biosensor for the detection of host and parasitic exosomes. (**a**) Figure reproduced with permission from [90], © 2018 Plos one, (**b**) Figure reproduced with permission from [91], © 2018 American Chemical Society.

Diverse nanostructures have been used for signal amplification of SPR immunosensors. Particularly, Au nanostructures have shown higher sensitivity to local refractive index variation near the Au nanosurface. Im et al. developed periodic Au nanohole arrays for label-free, high-throughput analysis of exosomes derived from ovarian cancer cells [87]. The Au nanoholes designed for a probing depth below 200 nm, can be easily matched to exosome size (below 100 nm) for highly sensitive detection. Moreover, the transmission setup allows for miniaturization of the system and tightly packed sensing arrays for the easy application of field diagnostic tests. Therefore, this SPR-based immunosensor is readily scalable for parallel measurements up to 10^5^ sensing spots. Thakur et al. described random arrays of self-assembled Au nanoislands for mass-produced sensitive and low-cost LSPR biosensors for tumor-related exosomes [88]. The advantage of this sensing system is the ability to distinguish exosomes from multivesicular vesicles (MVs) isolated from A-549, SH-SY5Y cells, blood serum, and urine in a lung cancer mouse model. This sensor could detect exosome concentrations ranging from 0.194 to 100 μg/mL. Bathini et al. detailed an Au nanoisland-assisted exosome detection platform with a streptavidin-biotin-polyethylene glycol (PEG)-venceremin (Vn96) complex [89]. Vn96, is a synthetic peptide, having a high affinity for heat shock proteins (HSPs) on the surface of exosomes. Each of the nanoislands can capture nine exosomes, meaning that the developed Au nanoisland platform can capture a much higher number of extracellular vesicles, thus offering a wide detection range from early stages to advanced stages of cancer. Wang et al. described a 30 nm-sized dual Au nanoparticle-assisted SPR immunosensor for the sensitive detection of exosomes released from MCF-7 breast cancer cells [86] (Figure 7b). The aptamer/T_30_-linked Au nanoparticles were bound to the target exosomes and A_30_-coated Au nanoparticles could be captured on the aptamer/T_30_-linked Au nanoparticles through A-T hybridization. Therefore, target exosomes could be detected at concentrations as low as 5 × 10^3^/mL by dual amplification between Au plate-Au nanoparticle and Au-Au nanoparticle. Raghu et al. discussed Au nanoplasmonic pillar arrays developed using nano- and micro-fabrication techniques for single exosome detection by an LSPR imaging analytical method [90] (Figure 8a). By sizing the individual nanopillar to approximately 100 nm, similar to the size of an exosome, it is possible to observe in situ single-exosome binding events in sub-femtomolar concentrations of exosomes via LSPR imaging signal. This approach results in a three orders of magnitude improvement of sensitivity over previously reported real-time, multiplexed platforms with an 80 nm Au-capped quartz nanopillar, which could minimize nonspecific binding events. 

Meanwhile, the development of microfluidics technologies provides novel exosome analysis platforms with excellent potential for the characterization of several diseases using a liquid biopsy without invasive methods. The microfluidic platforms have revealed great capacity for exosome analysis in clinical applications, including reduction of sample consumption, high-throughput analysis, reduction of cross-contamination, and the automation of isolation-detection to improve efficiency and reliability. Wang et al. reported that a microfluidic-integrated photonic crystal biosensor was successfully developed to distinguished host and parasitic exosomes released by the murine macrophage cell line J774A.1 and parasitic nematodes such as *Ascaris suum* [91] (Figure 8b). The surface of a photonic crystal was functionalized to anti-CD63, which could capture exosomes secreted by host cells. This biosensor exhibited a 2.18 × 10^9^ /mL LOD and is low-cost and disposable with a rapid assay time. The recent researches on SPR and LSPR-based analytical methods for exosomes detection are compared in Table 4.

## 6. Future Perspective and Conclusions

One of the final goals of the biosensors is the development of point-of-care testing (POCT) system for the prompt and precise therapy. In order that SPR immunosensors can be reached on the criteria for the point-of-care system, there should be integrated technologies such as portable platform, disposable chip, and miniaturization of the analytical machine. Recently, there has been presented a smartphone-integrated analytical system, enabling rapid diagnosis with connection to medical doctors and institutions. In addition, disposable-type chips have been also developed as a user convenience device with a simple operation such as color-change detection. On this wise, the SPR immunosensors have a great potential to utilize measurement of biomarkers due to their inherent label-free, cost-effective analysis with the rapid response time. Because of these advantages, SPR-based immunosensors will also facilitate the high-throughput and multiplex measurement of several biomarkers with the integration of the microfluidic system. On the other hand, the sensitivity is not high enough for the measurement of biomarkers in small volumes of body fluid, and it is not an intuitive color-change based method, with the exception of that based on Au nanoparticles [20,24]. Therefore, there have been several attempts to improve the sensitivity, that has been improved using magnetic activity, meta-surfaces, grating or photonic crystals [13,21,22,92]. Meanwhile, the plasmonic effect, which induces SPR phenomena, can induce other phenomena that can be applied to the development of immunosensors, including surface-enhanced Raman scattering (SERS), fluorescence resonance energy transfer (FRET), and metal-enhanced fluorescence (MEF). If the SERS, FRET, MEF-based analytical methods could be integrated with an SPR-based immunosensor using plasmonic effects, targets could be better measured and the shortcomings of each analytical method could be complemented [7,93,94,95,96,97]. Various plasmonic-based analytical methods each have advantages with noble metals and nanostructures, and it could be possible to develop higher performance immunosensors using an integrated platform. 

In this review, we introduced recent developments of SPR-based immunosensors with noble metals and nanostructures for the improvement of functionalities as efficient biosensors. The great advantage of this analytical method is in situ, label-free detection. It could result in the development of an immunosensor capable of measuring a target in a rapid, simple, and cost-effective manner. Currently, there are considerable shortcomings in SPR-based immunosensor systems pertaining to the challenges of highly sensitive detection. However, the integration of a plasmonic-based sensing system will offer a breakthrough platform for developing effective immunosensors for early diagnosis and POCT of various diseases, which, in turn, can improve biomedical, pharmaceutical, and clinical applications.

## Figures and Tables

**Table 1:** Comparison of SPR and LSPR-based immunosensors for protein biomarker detection.

Method	Working Principle	Target	Correlation Range	Detection Limit	Ref
PSPR	Site-directed antibody immobilization based on protein A/G	C-reactive protein (CRP)	1.2~80 ng/mL	1.2 ng/mL	[27]
	Antibody immobilization based on EDC/NHS coulping	metalloproteinases-9 (MMP-9)	10~200 ng/mL	8 pg/mL	[28]
	Site-directed antibody immobilization based on protein G	human growth hormone (hGH)	66.4~199 ng/mL *	21.9 ng/mL *	[29]
	Sandwich assay based on AuNP-antibody conjugate	Carcinoembryonic Antigen (CEA)	0~2.5 ng/mL	17.8 pg/mL	[32]
	Sandwich assay based on MWCNT-antibody conjugate	Tau Protein	9.87~78.9 ng/mL *	9.87 ng/mL *	[30]
	Sandwich assay based on AuNP-antibody conjugate	Cardiac troponin T (cTnT)	0.5~40 ng/mL	0.5 ng/mL	[33]
LSPR	nanorods (GNR) functionalized with poly (N-isopropylacrylamide) (PNIPAAM)	Troponin-T (TnT)	7.6~9.1 × 10^11^ fg/mL	8.4 fg/mL	[34]
	LSPR band shift based on interparticle crosslinking	Staphylococcal enterotoxin A (SEA)	0~250 ng/mL	5 ng/mL	[35]
	Core/shell nanoparticle Au@AgNPs and Ag@AuNPs	Staphylococcal enterotoxin A (SEA)	0~500 ng/mL	5.4 ng/mL	[36]
	Single AuNP	α-fetoprotein (AFP)	69~6.9 × 10^7^ fg/mL *	6.28 pg/mL *	[37]
	Single AuNP	Carcinoembryonic antigen (CEA)	180~1.8 × 10^8^ fg/mL *	16.9 pg/mL *	[37]
	Single AuNP	Prostate specific antigen (PSA)	28.4~2.84 × 10^6^ fg/mL *	284 fg/mL *	[37]
	Au/Ag Bimetallic nanostructures modified with polydopamine films	Bovine serum albumin (BSA)	0.01~100 ng/mL	0.01 ng/mL	[38]
	LSPR band shift based on the GNR etching resulted by TMB reaction	Carcinoembryonic antigen (CEA)	0~60 ng/mL	2.5 ng/mL	[39]
	LSPR band shift based on the GNR etching resulted by TMB reaction	Prostate specific antigen (PSA)	0~1275 pg/mL	75 pg/mL	[39]

* Some Values have been recalculated to express in terms of mL to provide better comparisons.

**Table 2:** Comparison of SPR and LSPR-based immunosensors for viral agent detection.

Method	Working Principle	Target	Correlation Range	Detection Limit	Ref
PSPR	Generation of new antibody	Avian Influenza A H7N9 Virus	2.3 × 10^2^~2.3 × 10^5^ copies/mL	402 copies/mL	[47]
	Antibody immobilization based on neutravidin-biotin	Dengue Virus Serotype 2 & 3	Not stated	2 × 10^4^ particles/mL	[49]
LSPR	Electrochemically deposited AuNPs	Human immunodeficiency Virus	0~200 pg/mL	25 pg/mL	[51]
	hetero-assembled AuNP based on sandwich-immunoassay	Hepatitis B surface antigen	200~1.25 × 10^5^ fg/mL	200 fg/mL	[52]
	Tilted fiber grating coated with AuNP	Newcastle disease virus	100~1 × 10^6^ fg/mL	100 fg/mL	[50]

**Table 3:** Comparison of SPR and LSPR-based immunosensors for bacterial detection.

Method	Working Principle	Target	Correlation Range (CFU/mL)	Detection Limit (CFU/mL)	Ref
PSPR	Immobilization of antibody against recombinant outer membrane protein (anti-OmpW) of Vibrio cholerae on Au chip	*Vibrio Cholerae*	10^1^–10^5^	10	[65]
	Functionalization of plasma polymers (PPs) on Au surface for a stable immobilization of antibodies	*Salmonella Typhimurium*	10^5^–10^8^	10^5^	[66]
	Immobilization of antibody using microarray spotter and flowed the samples on the Au chip	*Salmonella Typhimurium*	5.14 × 10^6^–5.14 × 10^8^	2.1 × 10^6^	[67]
	Detection of unbounded anti-Campylobacter Jejuni by the anti-rabbit IgG on Au chip	*Campylobacter Jejuni*	5–5 × 10^7^	131 ± 4	[68]
	Measurement of precipitated 4-chloro-1-naphthol by the HRP-tagged anti-Salmonella	*Salmonella Typhimurium*	10^2^–10^6^	10^2^	[69]
	Light escaping from optic fiber due to immunocapture of Escherichia coli	*Escherichia coli O157:H7*	Not stated	1.5 × 10^3^	[73]
	Functionalization of MoS_2_ nanosheet and anti- Escherichia coli on Au-coated optical fiber	*Escherichia coli O157:H7*	1 × 10^4^–8 × 10^4^	94	[74]
LSPR	Color change by the tyramine-functionalized Au nanoparticles and catalases-functionalized polystyrene beads, immobilized by Escherichia coli	*Escherichia coli O157:H7*	5 × 10^1^–5 × 10^4^	50	[71]

**Table 4:** Comparison of SPR and LSPR-based immunosensors for exosome detection.

Method	Working Principle	Target	Correlation Range	Detection Limit	Ref
PSPR	Immobilization of spotted anti-CD81 and -GM1 on Au chip	Exosomes from diverse neuronal cells	1–10 µg/mL	Not stated	[84]
	Immobilization of anti-HER2 on Au chip for detection of breast cancer-derived exosome	Breast cancer–derived exosomes	0.83–3.31 × 10^7^/mL	0.83 × 10^7^/mL	[85]
LSPR	Functionalization of PEG and anti-CD63 on periodic Au nanohole	Ovarian cancer–derived exosomes	4.03 × 10^5^–1.32 × 10^9^/mL	4.03 × 10^5^/mL	[87]
	Immobilized anti-CD9 on self-assembly Au islands for exosome detection	Exosomes from A-549 and SH-SY5Y cells	0.194–100 μg/mL	0.194 μg/mL	[88]
	Venceremin-functionalized Au nanoparticles on Au islands for capture the exosomes	Breast cancer-derived exosomes	Not stated	9 /μm^2^	[89]
	Functionalization of anti-CD63 on nanopillar array	Breast cancer–derived exosomes	Not stated	1 × 10^5^/mL	[90]
	Line shaped-Au nanopatterns with immobilization of anti-CD63	Murine macrophage–derived exosomes	2 × 10^9^–2 × 10^11^/mL	2.18 × 10^9^/mL	[91]

## References

[1] Gong C., Leite M.S. (2016). Noble Metal Alloys for Plasmonics. Acs Photonics.

[2] Haes A.J., Zou S.L., Schatz G.C., Van Duyne R.P. (2004). Nanoscale optical biosensor: Short range distance dependence of the localized surface plasmon resonance of noble metal nanoparticles. J. Phys. Chem B.

[3] Sugawa K., Tahara H., Yamashita A., Otsuki J., Sagara T., Harumoto T., Yanagida S. (2015). Refractive Index Susceptibility of the Plasmonic Palladium Nanoparticle: Potential as the Third Plasmonic Sensing Material. Acs Nano.

[4] Haes A.J., Zou S.L., Schatz G.C., Van Duyne R.P. (2004). A nanoscale optical biosensor: The long range distance dependence of the localized surface plasmon resonance of noble metal nanoparticles. J. Phys. Chem B.

[5] Schasfoort R.B. (2017). Handbook of surface plasmon resonance.

[6] Rich R.L., Myszka D.G. (2000). Advances in surface plasmon resonance biosensor analysis. Curr Opin Biotechnol.

[7] Yang L., Lee J.H., Rathnam C., Hou Y., Choi J.W., Lee K.B. (2019). Dual-Enhanced Raman Scattering-Based Characterization of Stem Cell Differentiation Using Graphene-Plasmonic Hybrid Nanoarray. Nano Lett.

[8] Eustis S., El-Sayed M.A. (2006). Why gold nanoparticles are more precious than pretty gold: Noble metal surface plasmon resonance and its enhancement of the radiative and nonradiative properties of nanocrystals of different shapes. Chem. Soc. Rev..

[9] Jain P.K., Huang X., El-Sayed I.H., El-Sayad M.A. (2007). Review of some interesting surface plasmon resonance-enhanced properties of noble metal nanoparticles and their applications to biosystems. Plasmonics.

[10] Nguyen H.H., Park J., Kang S., Kim M. (2015). Surface plasmon resonance: a versatile technique for biosensor applications. Sensors.

[11] Brolo A.G. (2012). Plasmonics for future biosensors. Nature Photon..

[12] Belotelov V., Akimov I., Pohl M., Kalish A., Kasture S., Vengurlekar A., Gopal A., Kotov V., Yakovlev D., Zvezdin A. (2011). Intensity magnetooptical effect in magnetoplasmonic crystals. Proceedings of Journal of Physics: Conference Series.

[13] Rizal C., Belotelov V. (2019). Sensitivity comparison of surface plasmon resonance (SPR) and magneto-optic SPR biosensors. Eur. Phys. J. Plus.

[14] Singh P. (2016). SPR Biosensors: Historical Perspectives and Current Challenges. Sensor Actuat. B-Chem..

[15] Kim D., Choi E., Lee C., Choi Y., Kim H., Yu T., Piao Y. (2019). Highly sensitive and selective visual detection of Cr(VI) ions based on etching of silver-coated gold nanorods. Nano. Converg..

[16] Rizal C., Pisana S., Hrvoic I. (2018). Improved magneto-optic surface plasmon resonance biosensors. Proceedings of Photonics.

[17] Rizal C., Pisana S., Hrvoic I., Fullerton E.E. (2018). Microstructure and magneto-optical surface plasmon resonance of Co/Au multilayers. J. Phys. Commun..

[18] Gupta B.D., Kant R. (2018). [INVITED] Recent advances in surface plasmon resonance based fiber optic chemical and biosensors utilizing bulk and nanostructures. Opt. Laser Technol..

[19] Masson J.F. (2017). Surface Plasmon Resonance Clinical Biosensors for Medical Diagnostics. ACS Sens..

[20] Piliarik M., Homola J. (2009). Surface plasmon resonance (SPR) sensors: approaching their limits?. Optics express..

[21] Rippa M., Castagna R., Pannico M., Musto P., Zyss J., Petti L. Multi-sensing meta nanostructures with surface-enhanced Raman scattering and surface plasmon resonance functionalities. Proceedings of the 19th Italian National Conference on Photonic Technologies (Fotonica 2017).

[22] Hasan M.R., Akter S., Rifat A.A., Rana S., Ahmed K., Ahmed R., Subbaraman H., Abbott D. (2017). Spiral photonic crystal fiber-based dual-polarized surface plasmon resonance biosensor. IEEE Sens. J..

[23] Cheng C., Chen H.-Y., Wu C.-S., Meena J.S., Simon T., Ko F.-H. (2016). A highly sensitive and selective cyanide detection using a gold nanoparticle-based dual fluorescence–colorimetric sensor with a wide concentration range. Sens. Actuators B Chem..

[24] Mauriz E., Garcia-Fernandez M.C., Lechuga L.M. (2016). Towards the design of universal immunosurfaces for SPR-based assays: A review. Trac-Trend Anal. Chem..

[25] Guo T., Gonzalez-Vila A., Loyez M., Caucheteur C. (2017). Plasmonic Optical Fiber-Grating Immunosensing: A Review. Sensors.

[26] Sahu V., Gupta A., Kumar R., Gupta T., Mohan A., Dey S. (2016). Quantification of Rac1 and Rac1b in serum of non small cell lung cancer by label free real time assay. Clin. Chim. Acta.

[27] Vashist S.K., Schneider E.M., Luong J.H. (2015). Surface plasmon resonance-based immunoassay for human C-reactive protein. Analyst.

[28] Mohseni S., Moghadam T.T., Dabirmanesh B., Jabbari S., Khajeh K. (2016). Development of a label-free SPR sensor for detection of matrixmetalloproteinase-9 by antibody immobilization on carboxymethyldextran chip. Biosen.s Bioelectron..

[29] Makaraviciute A., Ramanavicius A., Ramanaviciene A. (2015). Development of a reusable protein G based SPR immunosensor for direct human growth hormone detection in real samples. Anal. Methods.

[30] Lisi S., Scarano S., Fedeli S., Pascale E., Cicchi S., Ravelet C., Peyrin E., Minunni M. (2017). Toward sensitive immuno-based detection of tau protein by surface plasmon resonance coupled to carbon nanostructures as signal amplifiers. Biosens. Bioelectron..

[31] Kausaite-Minkstimiene A., Ramanaviciene A., Kirlyte J., Ramanavicius A. (2010). Comparative study of random and oriented antibody immobilization techniques on the binding capacity of immunosensor. Anal. Chem..

[32] Ermini M.L., Chadtova Song X., Springer T., Homola J. (2019). Peptide Functionalization of Gold Nanoparticles for the Detection of Carcinoembryonic Antigen in Blood Plasma via SPR-Based Biosensor. Front. Chem..

[33] Pawula M., Altintas Z., Tothill I.E. (2016). SPR detection of cardiac troponin T for acute myocardial infarction. Talanta.

[34] Ashaduzzaman M., Deshpande S.R., Murugan N.A., Mishra Y.K., Turner A.P., Tiwari A. (2017). On/off-switchable LSPR nano-immunoassay for troponin-T. Sci. Rep..

[35] Ben Haddada M., Hu D., Salmain M., Zhang L., Peng C., Wang Y., Liedberg B., Boujday S. (2017). Gold nanoparticle-based localized surface plasmon immunosensor for staphylococcal enterotoxin A (SEA) detection. Anal. Bioanal. Chem..

[36] Loiseau A., Zhang L., Hu D., Salmain M., Mazouzi Y., Flack R., Liedberg B., Boujday S. (2019). Core-Shell Gold/Silver Nanoparticles for Localized Surface Plasmon Resonance-Based Naked-Eye Toxin Biosensing. ACS Appl Mater. Interfaces.

[37] Lee J.U., Nguyen A.H., Sim S.J. (2015). A nanoplasmonic biosensor for label-free multiplex detection of cancer biomarkers. Biosens. Bioelectron..

[38] Jia K., Khayway M., Li Y., Bijeon J.-L., Adam P.-M., Déturche R., Guelorget B., François M., Louarn G., Ionescu R.E. (2014). Strong improvements of LSPR sensitivity by using Au/Ag bi-metallic nanostructures modified with poly-dopamine films. ACS Appl. Mater. Interfaces.

[39] Ma X., Lin Y., Guo L., Qiu B., Chen G., Yang H.H., Lin Z. (2017). A universal multicolor immunosensor for semiquantitative visual detection of biomarkers with the naked eyes. Biosens. Bioelectron..

[40] Zhang Z., Wang H., Chen Z., Wang X., Choo J., Chen L. (2018). Plasmonic colorimetric sensors based on etching and growth of noble metal nanoparticles: Strategies and applications. Biosens. Bioelectron..

[41] Inci F., Tokel O., Wang S., Gurkan U.A., Tasoglu S., Kuritzkes D.R., Demirci U. (2013). Nanoplasmonic quantitative detection of intact viruses from unprocessed whole blood. ACS Nano.

[42] Akira S., Uematsu S., Takeuchi O. (2006). Pathogen recognition and innate immunity. Cell.

[43] Feldmann H., Geisbert T.W. (2011). Ebola haemorrhagic fever. Lancet.

[44] World Health Organization (2014). Dengue and Severe Dengue.

[45] Lee J.H., Oh B.K., Choi J.W. (2015). Development of a HIV-1 Virus Detection System Based on Nanotechnology. Sensors.

[46] Caygill R.L., Blair G.E., Millner P.A. (2010). A review on viral biosensors to detect human pathogens. Anal. Chim. Acta.

[47] Chang Y.F., Wang W.H., Hong Y.W., Yuan R.Y., Chen K.H., Huang Y.W., Lu P.L., Chen Y.H., Chen Y.A., Su L.C. (2018). Simple Strategy for Rapid and Sensitive Detection of Avian Influenza A H7N9 Virus Based on Intensity-Modulated SPR Biosensor and New Generated Antibody. Anal. Chem.

[48] Jahanshahi P., Zalnezhad E., Sekaran S.D., Adikan F.R. (2014). Rapid immunoglobulin M-based dengue diagnostic test using surface plasmon resonance biosensor. Sci. Rep..

[49] Loureiro F.C.C.L., Neff H., Melcher E.U.K., Roque R.A., de Figueiredo R.M.P., Thirstrup C., Borre M.B., Lima A.M.N. (2017). Simplified immunoassay for rapid Dengue serotype diagnosis, revealing insensitivity to non-specific binding interference. Sensing and Bio-Sensing Research.

[50] Luo B., Xu Y., Wu S., Zhao M., Jiang P., Shi S., Zhang Z., Wang Y., Wang L., Liu Y. (2018). A novel immunosensor based on excessively tilted fiber grating coated with gold nanospheres improves the detection limit of Newcastle disease virus. Biosens. Bioelectron..

[51] Lee J.H., Kim B.C., Oh B.K., Choi J.W. (2013). Highly sensitive localized surface plasmon resonance immunosensor for label-free detection of HIV-1. Nanomedicine.

[52] Kim J., Oh S.Y., Shukla S., Hong S.B., Heo N.S., Bajpai V.K., Chun H.S., Jo C.H., Choi B.G., Huh Y.S. (2018). Heteroassembled gold nanoparticles with sandwich-immunoassay LSPR chip format for rapid and sensitive detection of hepatitis B virus surface antigen (HBsAg). Biosens. Bioelectron..

[53] Salyers A.A., Whitt D.D., Whitt D.D. (1994). Bacterial Pathogenesis: A Molecular Approach.

[54] Park Y.M., Lim S.Y., Jeong S.W., Song Y., Bae N.H., Hong S.B., Choi B.G., Lee S.J., Lee K.G. (2018). Flexible nanopillar-based electrochemical sensors for genetic detection of foodborne pathogens. Nano Converg..

[55] Alsan M., Schoemaker L., Eggleston K., Kammili N., Kolli P., Bhattacharya J. (2015). Out-of-pocket health expenditures and antimicrobial resistance in low-income and middle-income countries: an economic analysis. Lancet. Infect. Dis..

[56] Adegbola R.A., DeAntonio R., Hill P.C., Roca A., Usuf E., Hoet B., Greenwood B.M. (2014). Carriage of Streptococcus pneumoniae and other respiratory bacterial pathogens in low and lower-middle income countries: a systematic review and meta-analysis. PLoS ONE.

[57] Kim D.W., Chun H.J., Kim J.H., Yoon H., Yoon H.C. (2019). A non-spectroscopic optical biosensor for the detection of pathogenic Salmonella Typhimurium based on a stem-loop DNA probe and retro-reflective signaling. Nano. Converg..

[58] Lazcka O., Del Campo F.J., Munoz F.X. (2007). Pathogen detection: a perspective of traditional methods and biosensors. Biosens. Bioelectron..

[59] Mansfield L., Forsythe S. (2000). The detection of Salmonella using a combined immunomagnetic separation and ELISA end-detection procedure. Lett. Appl. Microbiol..

[60] Cho I.H., Irudayaraj J. (2013). In-situ immuno-gold nanoparticle network ELISA biosensors for pathogen detection. Int. J. Food Microbiol..

[61] Torun O., Hakki Boyaci I., Temur E., Tamer U. (2012). Comparison of sensing strategies in SPR biosensor for rapid and sensitive enumeration of bacteria. Biosens. Bioelectron..

[62] Ahmed A., Rushworth J.V., Hirst N.A., Millner P.A. (2014). Biosensors for whole-cell bacterial detection. Clin. Microbiol. Rev..

[63] Oh B.K., Lee W., Chun B.S., Bae Y.M., Lee W.H., Choi J.W. (2005). The fabrication of protein chip based on surface plasmon resonance for detection of pathogens. Biosens. Bioelectron..

[64] Jyoung J.Y., Hong S.H., Lee W., Choi J.W. (2006). Immunosensor for the detection of Vibrio cholerae O1 using surface plasmon resonance. Biosens. Bioelectron..

[65] Taheri R.A., Rezayan A.H., Rahimi F., Mohammadnejad J., Kamali M. (2016). Development of an immunosensor using oriented immobilized anti-OmpW for sensitive detection of Vibrio cholerae by surface plasmon resonance. Biosens. Bioelectron..

[66] Makhneva E., Farka Z., Skladal P., Zajickova L. (2018). Cyclopropylamine plasma polymer surfaces for label-free SPR and QCM immunosensing of Salmonella. Sensor Actuat. B-Chem..

[67] Chen J., Park B. (2018). Label-free screening of foodborne Salmonella using surface plasmon resonance imaging. Anal. Bioanal. Chem..

[68] Masdor N.A., Altintas Z., Shukor M.Y., Tothilll I.E. (2019). Subtractive inhibition assay for the detection of Campylobacter jejuni in chicken samples using surface plasmon resonance. Scientific Reports.

[69] Farka Z., Jurik T., Pastucha M., Skladal P. (2016). Enzymatic Precipitation Enhanced Surface Plasmon Resonance Immunosensor for the Detection of Salmonella in Powdered Milk. Anal. Chem..

[70] Zou F., Wang X.X., Qi F.J., Kohn K., Lee J., Zhou H.J., Chen H.X. (2017). Magneto-plamonic nanoparticles enhanced surface plasmon resonance TB sensor based on recombinant gold binding antibody. Sensor Actuat. B-Chem..

[71] Zheng L., Cai G., Wang S., Liao M., Li Y., Lin J. (2019). A microfluidic colorimetric biosensor for rapid detection of Escherichia coli O157: H7 using gold nanoparticle aggregation and smart phone imaging. Biosens. Bioelectron..

[72] Zhang L., Salmain M.l., Liedberg B., Boujday S. (2019). Naked Eye Immunosensing of Food Biotoxins Using Gold Nanoparticle-Antibody Bioconjugates. ACS Appl. Nano Mater..

[73] Arcas A.D.S., Dutra F.D.S., Allil R., Werneck M.M. (2018). Surface Plasmon Resonance and Bending Loss-Based U-Shaped Plastic Optical Fiber Biosensors. Sensors.

[74] Kaushik S., Tiwari U.K., Pal S.S., Sinha R.K. (2019). Rapid detection of Escherichia coli using fiber optic surface plasmon resonance immunosensor based on biofunctionalized Molybdenum disulfide (MoS2) nanosheets. Biosens. Bioelectron..

[75] Mulcahy L.A., Pink R.C., Carter D.R. (2014). Routes and mechanisms of extracellular vesicle uptake. J. Extracell Vesicles.

[76] Witwer K.W., Buzas E.I., Bemis L.T., Bora A., Lasser C., Lotvall J., Nolte-’t Hoen E.N., Piper M.G., Sivaraman S., Skog J. (2013). Standardization of sample collection, isolation and analysis methods in extracellular vesicle research. J. Extracell Vesicles.

[77] Wiklander O.P., Nordin J.Z., O’Loughlin A., Gustafsson Y., Corso G., Mäger I., Vader P., Lee Y., Sork H., Seow Y. (2015). Extracellular vesicle in vivo biodistribution is determined by cell source, route of administration and targeting. J. Extracell Vesicles.

[78] Bu J., Shim J.E., Lee T.H., Cho Y.H. (2019). Multi-modal liquid biopsy platform for cancer screening: screening both cancer-associated rare cells and cancer cell-derived vesicles on the fabric filters for a reliable liquid biopsy analysis. Nano Converg..

[79] Zhang J., Li S., Li L., Li M., Guo C., Yao J., Mi S. (2015). Exosome and exosomal microRNA: trafficking, sorting, and function. Genomics Proteomics Bioinformatics.

[80] Théry C., Zitvogel L., Amigorena S. (2002). Exosomes: composition, biogenesis and function. Nat. Rev. Immunol..

[81] Alderton G.K. (2012). Exosomes drive premetastatic niche formation. Nat. Rev. Cancer.

[82] Azmi A.S., Bao B., Sarkar F.H. (2013). Exosomes in cancer development, metastasis, and drug resistance: a comprehensive review. Cancer Metastasis Rev..

[83] Brinton L.T., Sloane H.S., Kester M., Kelly K.A. (2015). Formation and role of exosomes in cancer. Cell Mol. Life Sci..

[84] Picciolini S., Gualerzi A., Vanna R., Sguassero A., Gramatica F., Bedoni M., Masserini M., Morasso C. (2018). Detection and Characterization of Different Brain-Derived Subpopulations of Plasma Exosomes by Surface Plasmon Resonance Imaging. Anal. Chem.

[85] Sina A.A.I., Vaidyanathan R., Wuethrich A., Carrascosa L.G., Trau M. (2019). Label-free detection of exosomes using a surface plasmon resonance biosensor. Anal. Bioanal. Chem..

[86] Wang Q., Zou L., Yang X., Liu X., Nie W., Zheng Y., Cheng Q., Wang K. (2019). Direct quantification of cancerous exosomes via surface plasmon resonance with dual gold nanoparticle-assisted signal amplification. Biosens. Bioelectron..

[87] Im H., Shao H., Park Y.I., Peterson V.M., Castro C.M., Weissleder R., Lee H. (2014). Label-free detection and molecular profiling of exosomes with a nano-plasmonic sensor. Nat. Biotechnol.

[88] Thakur A., Qiu G., Ng S.P., Guan J., Yue J., Lee Y., Wu C.L. (2017). Direct detection of two different tumor-derived extracellular vesicles by SAM-AuNIs LSPR biosensor. Biosens. Bioelectron..

[89] Bathini S., Raju D., Badilescu S., Kumar A., Ouellette R.J., Ghosh A., Packirisamy M. (2018). Nano-Bio Interactions of Extracellular Vesicles with Gold Nanoislands for Early Cancer Diagnosis. Research (Wash D C).

[90] Raghu D., Christodoulides J.A., Christophersen M., Liu J.L., Anderson G.P., Robitaille M., Byers J.M., Raphael M.P. (2018). Nanoplasmonic pillars engineered for single exosome detection. PLoS ONE.

[91] Wang Y.F., Yuan W., Kimber M., Lu M., Dong L. (2018). Rapid Differentiation of Host and Parasitic Exosome Vesicles Using Microfluidic Photonic Crystal Biosensor. Acs Sens..

[92] Kotlarek D., Vorobii M., Ogieglo W., Knoll W., Rodriguez-Emmenegger C., Dostalek J. (2019). Compact Grating-Coupled Biosensor for the Analysis of Thrombin. ACS Sens..

[93] Cong S., Yuan Y., Chen Z., Hou J., Yang M., Su Y., Zhang Y., Li L., Li Q., Geng F. (2015). Noble metal-comparable SERS enhancement from semiconducting metal oxides by making oxygen vacancies. Nat. Commun..

[94] Choi J.H., El-Said W.A., Choi J.-W. (2019). Highly sensitive surface-enhanced Raman spectroscopy (SERS) platform using core/double shell (Ag/polymer/Ag) nanohorn for proteolytic biosensor. Appl. Surf. Sci..

[95] El-Said W.A., Yoon J., Choi J.W. (2018). Nanostructured surfaces for analysis of anticancer drug and cell diagnosis based on electrochemical and SERS tools. Nano Converg..

[96] Lee J.H., Choi J.H., Chueng S.D., Pongkulapa T., Yang L., Cho H.Y., Choi J.W., Lee K.B. (2019). Nondestructive Characterization of Stem Cell Neurogenesis by a Magneto-Plasmonic Nanomaterial-Based Exosomal miRNA Detection. ACS Nano.

[97] Mohammadi S., Salimi A., Hamd-Ghadareh S., Fathi F., Soleimani F. (2018). A FRET immunosensor for sensitive detection of CA 15-3 tumor marker in human serum sample and breast cancer cells using antibody functionalized luminescent carbon-dots and AuNPs-dendrimer aptamer as donor-acceptor pair. Anal. Biochem..

